# Site-Specific Immunosuppression in Vascularized Composite Allotransplantation: Prospects and Potential

**DOI:** 10.1155/2013/495212

**Published:** 2013-02-13

**Authors:** Jonas T. Schnider, Matthias Weinstock, Jan A. Plock, Mario G. Solari, Raman Venkataramanan, Xin Xiao Zheng, Vijay S. Gorantla

**Affiliations:** ^1^Department of Plastic and Reconstructive Surgery, University of Pittsburgh School of Medicine, Pittsburgh, PA 15261, USA; ^2^Disciplina de Cirurgia Plástica, Escola Paulista de Medicina, Universidade Federal de São Paulo, São Paulo 04024-900, Brazil; ^3^Division of Plastic and Hand Surgery, University Hospital Zurich, Raemistrasse 100, CH-8091 Zurich, Switzerland; ^4^Department of Pharmaceutical Sciences, University of Pittsburgh School of Pharmacy, Pittsburgh, PA 15261, USA; ^5^Research Center for Translational Medicine, Shanghai East Hospital, Tongji University, Shanghai 200120, China; ^6^Pittsburgh Reconstructive Transplantation Program, Department of Plastic Surgery, 3550 Terrace Street, Pittsburgh, PA 15261, USA

## Abstract

Skin is the most immunogenic component of a vascularized composite allograft (VCA) and is the primary trigger and target of rejection. The skin is directly accessible for visual monitoring of acute rejection (AR) and for directed biopsy, timely therapeutic intervention, and management of AR. Logically, antirejection drugs, biologics, or other agents delivered locally to the VCA may reduce the need for systemic immunosuppression with its adverse effects. Topical FK 506 (tacrolimus) and steroids have been used in clinical VCA as an adjunct to systemic therapy with unclear beneficial effects. However, there are no commercially available topical formulations for other widely used systemic immunosuppressive drugs such as mycophenolic acid, sirolimus, and everolimus. Investigating the site-specific therapeutic effects and efficacy of systemically active agents may enable optimizing the dosing, frequency, and duration of overall immunosuppression in VCA with minimization or elimination of long-term drug-related toxicity.

## 1. Introduction

Since 1998, more than 200 patients have received vascularized composite allografts (VCAs). VCAs such as hand, face, or abdominal wall transplants are unique from solid organ transplants (SOTs) because of their heterogenous tissue composition that may include skin, muscle, vessels, tendon, nerve, lymph nodes, cartilage, bone, and bone marrow. Importantly, skin has been shown to be the most immunogenic constituent of certain VCA [1] mandating long-term immunosuppression for graft survival. However, transplantation of a whole limb allograft interestingly elicits a lower immune response than transplantation of individual tissue components, such as skin in the form of vascularized or nonvascularized grafts [1].

Despite evolving clinical experience and progress in the understanding of the biology of VCA, one of the main factors preventing wider acceptance or routine clinical application is the associated adverse effects of long-term immunosuppression. Antirejection therapy can lead to diabetes mellitus, nephrotoxicity, osteonecrosis, leukopenia, hypertension, hyperlipidemia, and opportunistic bacterial and viral infections [2, 3]. Since VCAs are non-life-saving procedures, the risks and toxicity of immunosuppression must be carefully balanced against their potential life enhancing benefits in recipients. 

Unlike most SOTs, VCAs offer unique opportunities for local delivery of immunosuppressive medications directly to the graft. The rationale for such site-specific, transplant-targeted delivery of immunosuppression is to reduce systemic exposure and global collateral or end-organ adverse effects. Hypothetically, site-specific graft immunosuppression could facilitate minimization of overall dosing, frequency, and duration of systemic immunosuppression and also help reduce the number of systemic drugs required for desired efficacy and improved graft survival. 

Like in SOT, noncompliance with immunosuppressive medication is emerging as an imminent threat to long-term graft survival in clinical VCA. There has been at least one report of a VCA loss (1 hand has been explanted) due to confirmed medication noncompliance [4]. It may be argued that reducing the overall number or frequency of administration of systemic agents for graft maintenance by adjunctive use of site-specific graft immunosuppression may improve compliance with oral medications. The VCA graft is the trigger and the target of the recipient immune response. Advances in systemic immunosuppression have been based on insights into key pathways in donor or recipient allorecognition including adaptive or innate responses. Based on the specific cellular and molecular targets and mechanisms of action of various immunosuppressive agents, it may be expected that different agents (used topically or systemically) affect different pathways, components, or cascades of the immune response after VCA. In some cases, this may result in more specific immunosuppression, whereas in others it may result in diversion of the immune response from the allograft while preserving systemic immunity. Such innovation in maintenance immunosuppression has resulted in dramatic reductions in AR and improvement in short- and long-term patient and graft outcomes in SOT. Although true tolerance is still a holy grail, the reduction or elimination of some immunosuppressive agents as part of multidrug regimens with their long-term toxicities is a potential near term achievable goal. Such a goal could be realized in VCA by graft delivered therapies used adjunctively with systemic drugs. Site-specific delivery of agents that individually and selectively inhibit the immune trigger processes such as APC activation, T-cell priming, B-cell help, transendothelial migration of activated T or B cells, and regional lymph nodal mechanisms including early or ongoing antigen presentation could enable reductions in need for systemic immunosuppression by complementary, synergistic, or additive effects. Local agents may also prevent ischemia-reperfusion injury, in which leukocyte-endothelial cell interactions (as in rejection) are thought to play a key role. Optimal graft tissue concentrations/bioavailability of locally delivered drugs (mono or combination therapy) may indeed allow rejection control with lower systemic troughs compared to similar antirejection efficacy with higher overall systemic troughs secondary to mono- or multidrug maintenance therapy.

## 2. Route of Application

Several strategies exist for local delivery of immunosuppressive/immunomodulatory therapies in VCAs: (1) topical therapies are applied to the surface of the skin but do not effectively overcome the skin barrier, for example, therapies for psoriasis or atopic dermatitis; (2) transdermal therapies such as patches are more successful in overcoming the skin barrier and promote a local or systemic effect; (3) subcutaneous or intradermal (e.g., insulin injection, TB vaccination); and (4) intravascular delivery of the immunosuppressive drugs directly into the transplanted allograft [5]. In VCA, all of these strategies might be of potential interest and of significant relevance, depending on the immunosuppressive regimen and the predicated clinical situation. They can be also considered as adjunct or alternative to systemic treatment [6]. 

It may be a challenge to distinguish the unique effects of topical versus transdermal therapy. In certain cases, topical delivery can result in systemic levels that are higher or lower than expected. Topical calcineurin inhibitors and glucocorticoids have been the focus of several such studies. However, conclusions from these and other similar studies are limited by confounding effects of increased systemic absorption of the locally applied agents (due to locally inflamed or abraded skin) or failure to routinely monitor blood levels or the drug toxicity [7]. In studies with topical cyclosporine, low systemic levels have been reported [8].

### 2.1. Topical Therapy

The classical formulations used for topical therapy are ointments, crèmes, lotions, gels, and powders, which are widely used in the treatment of dermatological disorders. Topical calcineurin inhibitors were initially developed for the treatment of atopic dermatitis [9]. FDA-approved commercial formulations available include tacrolimus (Protopic), which is available as an ointment, pimecrolimus (Elidel), which is a cream, and clobetasol propionate, which is also a cream. Different reports suggest a beneficial effect in conditions such as seborrheic dermatitis, vitiligo, atopic dermatitis, lichen planus, or psoriasis [10–12]. 

Sirolimus (rapamycin) is a macrolide antibiotic, structurally similar to tacrolimus. It binds to FKBP-12 and affects the G_1_ phase of the cell cycle by acting on a unique cellular target called mammalian target of rapamycin (mTOR) [13]. Sirolimus has been mainly used systemically in SOT, but topical treatment has also been reported in an experimental/preclinical setting in dermatology. It has been shown to have beneficial effects in conditions such as facial angiofibromas, psoriasis, and lichen planus, with either undetectable or insignificant blood levels [14–16]. Sirolimus lotion [16] has been used in oral lichen planus.

Mycophenolate mofetil (MMF) is an ester of mycophenolic acid (MPA). It selectively blocks proliferation of T cells and suppresses antibody formation by B cells [17]. There is only limited data that is available on topical MPA. Different MMF formulations as eye drop solutions and aqueous suspensions have been evaluated. In a corneal allograft model topical MPA showed no significant difference compared to vehicle only or no treatment [18]. However, Shoji et al. showed that topical MPA may be effective in an experimental model of allergic contact dermatitis [19]. None of the above-mentioned agents have been systematically evaluated as a topical therapy for VCA.

### 2.2. Transdermal Therapy

Transdermal patches that have been used for the delivery of drugs include but are not limited to nicotine, nitroglycerin, fentanyl, or estrogen. Different methods to optimize systemic exposure of other drugs have also been described. Prausnitz and Langer [20] proposed a categorization of transdermal delivery systems into three generations: first-generation transdermal delivery systems are used for the delivery of small, lipophilic, low-dose drugs and have been clinically implemented [20]. Drug transport relies on diffusion through the stratum corneum and the epidermis into the dermis. Second-generation delivery systems increase skin permeability and driving forces to improve systemic delivery. Different methods have been introduced such as chemical enhancement [21], noncavitational ultrasound and iontophoresis [22]. Third-generation delivery systems rely on disruption of the stratum corneum and include combinations of chemical enhancers, biochemical enhancers, transfollicular strategies, microneedles [23], thermal ablation, microdermabrasion, laser electroporation (microporation), and cavitational ultrasound [20]. In this context numerous different patents have also been reported. To our knowledge, none of these novel methods of transdermal application have been evaluated either in translational models or clinical settings involving VCA.

### 2.3. Intradermal/Subcutaneous Injections

Several agents (drugs, antibodies) have been evaluated in dermatology for therapeutic efficacy via the intradermal/subcutaneous administration. The intradermal route is the preferred route for some vaccinations (such as BCG), while subcutaneous delivery is superior for some therapies (such as insulin). Efomycine M is a novel small molecule that blocks E- and P-selectin [24]. It has shown therapeutic efficacy in mouse models of psoriasis [25]. Hautz et al. could significantly prolong hind limb allograft survival in rats with weekly subcutaneous injections of efomycine M with short-course systemic antilymphocyte serum (ALS) and tacrolimus [24]. Local intragraft delivery of other leucocyte migration blockers such as anti-ICAM-1, anti-LFA-1, and the fibrin derivative B *β* 15–42, which blocks VE-cadherin, has also been shown to prolong VCA survival with or without short-course systemic therapy.

### 2.4. Local Therapy

Local therapy aims at the utilization of drug administration systems to establish a more selective delivery of currently available nonspecific immunosuppressive agents directed at the vascular supply of the transplanted organ/graft through the spatial and temporal control of drug delivery, for example, via use of catheter delivery or Alzet miniosmotic pump systems [26]. 

## 3. Site-Specific Immunosuppression in Solid Organ Transplantation

Limited studies have evaluated the application of intravascular administration of immunosuppressive drugs in SOT [26–28], demonstrating a beneficial outcome of site-specific immunosuppression by delivering the immunosuppressive agents directly to the graft. Ruers et al. [29] introduced an implantable osmotic minipump delivering prednisolone with an arterial catheter to the renal allograft. Local application was superior to systemic application at a dosage of 4 mg/kg body weight per day, whereas i.p. or i.v. administration was ineffective at this dose. 

Yano et al. [30] compared the effects of tacrolimus administered via different routes (hepatic artery, portal vein, and systemic circulation) in a rat liver allotransplantation model. In addition to systemic application of tacrolimus (0.08–1.28 mg/kg) for 7 days, a low dose of tacrolimus (0.32 mg/kg) was infused into the hepatic artery or the portal vein for three days. In contrast to additional infusions of systemic tacrolimus, local delivery led to dramatically improved allograft outcomes. Further, local delivery of immunosuppressants was superior to systemic delivery in cardiac [31] and pancreatic islet cell [32] transplantation models.

The lung offers a unique opportunity of directed local application of drugs, as the highly antigenic airway epithelium of the bronchial/bronchiolar passages is readily accessible for drug delivery. Nebulized cyclosporine delivered via inhalation to the lung has been used to treat episodes of AR as well as chronic rejection (CR) in lung transplants at the University of Pittsburgh. The beneficial effect of local delivery has also been shown with aerosolized cyclosporine as a rescue therapy for refractory AR [33, 34]. A randomized trial over a two-year period showed lower rates of CR and improved survival of lung grafts, but no difference in the incidence of AR [35]. 

Inhaled cyclosporine is associated with airway irritation clinically, requiring the addition of lidocaine to the preparation. Tacrolimus is more potent then cyclosporine, thus allowing for similar or superior efficacy with twenty to forty times less drug. Deuse et al. [36] presented a study aiming at elucidating the mechanism of inhaled tacrolimus using in vivo and in vitro models. Human airway epithelium was grown in vitro at an air-liquid interface in order to simulate inhaled and systemic application of tacrolimus. The aerosolized tacrolimus was capable of inducing higher tissue concentrations and lower blood concentrations than the systemic application of tacrolimus. Concentrations of tacrolimus in tissue from the trachea and the lung showed higher peak values after 1 hour if the animals had received aerosol treatment compared with oral gavage. The peaks, however, were followed by a rapid decline in tissue drug concentrations, and 24-hour trough tissue levels were approximately 2 times lower after TAC inhalation. In vitro IL-1*β* was used to simulate an inflammation similar to AR or ischemia reperfusion. The inhibitory effects of tacrolimus on NF-*κ*B phosphorylation and nuclear translocation of human airway epithelial cells in the presence of IL-1*β* could be clearly demonstrated, and a reduction of inflammatory cytokine secretion was observed after aerosolized tacrolimus. Airway epithelium absorbed tacrolimus from the aerosol and enabled effective inhibition of subepithelial lymphocyte activation.

To our knowledge, there is only one report of intra-arterial administration of immunosuppressive drugs in humans. Furtado et al. [37] reported two patients who received local immunosuppression. The first patient suffered a small bowel rejection after combined liver and small bowel transplantation. At 3 months after the initial transplantation a second small bowel transplant was performed from a cadaveric ABO identical, two-HLA-match donor. A 15 cm segment of the middle colic artery was isolated in continuity with the superior mesenteric artery, allowing insertion of a catheter. The immunosuppressive treatment consisted of methylprednisolone and tacrolimus (0.12–0.06 mg/kg per day) via catheter for 3 weeks. The second patient received a small bowel transplant from a cadaveric ABO-identical, total HLA-mismatched donor. The catheter was inserted in the same way as mentioned into the middle colic artery and maintained for 7 weeks. Two major episodes of acute cellular rejections were treated with 250 mg boluses of methylprednisolone and elevated doses of tacrolimus (dosage 0.08–0.02 mg/kg per day). Both patients could be discharged from the hospital and continue work and education. These two cases illustrate that in very critical patients local intraarterial immunosuppressive treatment is feasible.

## 4. Site-Specific Immunosuppression in VCA

The skin is the largest organ in the body [1, 38] and is a physical and immunological barrier [39, 40]. It is the most immunogenic component of a VCA [1]. In hand transplantation the early signs of clinical rejection are seen in the skin. Pathologically, this may correlate with differential degrees of cellular infiltrates in the epidermal-dermal or adnexal structures as classified by the Banff system for VCA. [41, 42]. The phenomenon of “split tolerance” is associated with indefinite survival of the musculoskeletal portion, but rejection of the epidermis of VCA and has been reported in animal models [43]. It seems to be logical therefore to locally target the skin and avoid high degree of overall systemic immunosuppression [44]. However, the cumulative experience on the use of graft-delivered therapies in clinical VCA such as hand or face transplants is varied and inconsistent, leading to debate on the practical utility of such strategies.

Over 90 upper extremities and 20 face transplantations have been performed worldwide in the past decade [45]. Most AR episodes have been amenable to bolus systemic steroids, changes to systemic immunosuppression with or without interventions such as extracorporeal phototherapy and topical tacrolimus ointment, and/or steroid (clobetasol) creams used PRN as adjunctive therapy [45]. In hand transplantation, there have been reports of topical tacrolimus and clobetasol resulting in adequate to complete response during Banff Grade 1 and 1-2 rejection episodes [46]. Despite their frequent use, the efficacy of local immunosuppression currently remains unproven in clinical VCA, due to the challenge of designing carefully controlled studies or implementation of such treatment per established protocols that rely on clinicopathologic correlation [47].

In preclinical studies, topical tacrolimus has been shown to prolong graft survival in hind limb allotransplantation models as well as in face transplantation models [7, 48]. Topical tacrolimus did not lead to elevated blood concentrations, but a 100-fold higher concentration of the drug was observed in the skin versus underlying tissues [8]. Compared to topical steroids, which can result in collagen atrophy and skin thinning, tacrolimus has few local adverse effects [49]. Compared to cyclosporine, which has also been evaluated in topical treatment, tacrolimus has been shown to have superior effect due to its higher potency. Different mechanisms of action of topical tacrolimus and pimecrolimus have been suggested in the literature. There may be a depletion of inflammatory dendritic epidermal cells [50], or a reduction of costimulatory molecules on dendritic cells and alteration of their function [51, 52]. Additional mechanisms include apoptosis-induced depletion of T cells [53] and reduced expression of adhesion molecules [54].

Shirbacheh et al. [55–57] were the first to describe intraarterial delivery of immunosuppressive drugs in VCA. These pioneering studies involved calcineurin inhibitors in an experimental large animal VCA limb transplant model to correlate tissue and local pharmacokinetics with systemic trough levels and adverse effects [55, 58]. The conclusion was that tacrolimus is pharmacokinetically inferior to cyclosporine. Despite its demonstrated efficacy in experimental and clinical transplantation, our Findings suggest tacrolimus would not be an appropriate immunosuppressant to be delivered via the i.a. route for prevention of limb allograft rejection.

The monitoring of a clinical VCA such as a hand or face transplant has conventionally relied on protocol skin biopsies and those biopsies mandated by clinical signs of rejection. Deeper tissues (muscle, nerve, vessel and bone) have not been biopsied on a regular basis in VCA. Based on these paradigms, there are several questions that remain to be answered in VCA that could impact the overall relevance of topical immunosuppression in VCA.

1. What are the temporal kinetics and dynamics of rejection of the various tissue components of VCA after transplantation?
2. Is clinical and histopathologic resolution of rejection in skin (upon systemic or topical intervention) associated with similar outcome in underlying tissues?
3. Is continuous topical treatment superior to systemic therapy by augmenting local tissue concentrations at the trigger site of the immune response?

## 5. Development of New Topical Formulations: Promise and Potential

Tacrolimus and clobetasol are available for topical administration as they are commercially available with FDA approval for other indications. Other immunosuppressive drugs do have applications in dermatology and have only been used systemically, or in experimental skin formulations. With the intention to develop new immunosuppressive formulations, we started an in vitro study comparing novel formulations of topical MPA and sirolimus to commercially available tacrolimus and clobetasol preparations. Our goal was to evaluate the local release, tissue bioavailability, and pharmacokinetics of these immunosuppressive drugs. For the in vitro studies, a semipermeable membrane simulating the skin barrier was used in Franz Diffusion Cells. Preliminary results showed that less than 10% of the original dose of tacrolimus penetrates the membrane. In contrast, approximately 10% of the initial dose of sirolimus diffuses across the membrane. With MPA, 38% of the initial applied dose diffuses into the recipient compartment. This diffusion was time dependent and continued to occur past 24 hrs. Based on in vitro data, we hypothesize that if used at the right dosing and frequency, both sirolimus and tacrolimus could achieve good local concentrations in the skin, with lower levels in deeper tissues and minimal systemic drug exposure. MPA on the other hand appears to have the greatest potential for diffusion across the membrane in vitro. This suggests that topical application of MPA will result in higher concentrations of the drug in muscle and other local sites with possibly some increase in systemic concentrations as well. Controlling the concentration of MPA in the formulation and alterations in the frequency of dosing could further minimize systemic drug exposure after topical application of MPA. 

## 6. Conclusion

Graft-targeted and delivered site-specific immunosuppression is uniquely suited for VCA due to direct accessibility to such interventions. Local graft manipulation with targeted preloading of immunosuppression may also have potential benefit in supplementing or reducing the intensity of systemic induction therapy. Such a strategy may be customized to deliver high or sustained concentrations of drugs, antibodies, biologics, and other molecules, which may not be feasible via the systemic route due to short half-life, inactivation due to first pass hepatic metabolism, or enzymatic degradation. The additive, adjuvant, complementary, or synergistic role of locally administered immunosuppression in conjunction with systemic delivery cannot be underestimated in VCA with their accessible cutaneous or mucosal components amenable to such innovative delivery techniques. The minimization of need for multiple drugs, and their dosing, frequency, and duration of treatment with concomitant toxicity of such systemic therapy by site-specific therapies needs further investigation and collaborative inquiry especially in VCA which are not life-saving grafts such as SOT, but life-enhancing procedures. Potentially, targeting distinct mechanistic pathways and molecular targets in the skin immune system with combination of site-specific immunosuppression may facilitate an immunomodulatory environment that may indeed induce a permissive milieu for the development of tolerant VCA.

## References

[1] Lee WPA, Yaremchuk MJ, Pan YC, Randolph MA, Tan CM, Weiland AJ (1991). Relative antigenicity of components of a vascularized limb allograft. *Plastic and Reconstructive Surgery*.

[2] Schneeberger S, Lucchina S, Lanzetta M (2005). Cytomegalovirus-related complications in human hand transplantation. *Transplantation*.

[3] Lanzetta M, Petruzzo P, Margreiter R (2005). The international registry on hand and composite tissue transplantation. *Transplantation*.

[4] Breidenbach WC, Tobin GR, Gorantla VS, Gonzalez RN, Granger DK (2002). A position statement in support of hand transplantation. *Journal of Hand Surgery*.

[5] Gruber SA, Shirbacheh MV, Jones JW, Barker JH, Breidenbach WC (2000). Local drug delivery to composite tissue allografts.. *Microsurgery*.

[6] Gorantla VS, Brandacher G, Schneeberger S (2011). Favoring the risk-benefit balance for upper extremity transplantation—the Pittsburgh Protocol. *Hand Clinics*.

[7] Solari MG, Washington KM, Sacks JM (2009). Daily topical tacrolimus therapy prevents skin rejection in a rodent hind limb allograft model. *Plastic and Reconstructive Surgery*.

[8] Black KS, Nguyen DK, Proctor CM, Patel MP, Hewitt CW (1990). Site-specific suppression of cell-mediated immunity by cyclosporine. *Journal of Investigative Dermatology*.

[9] Luger T, Paul C (2007). Potential new indications of topical calcineurin inhibitors. *Dermatology*.

[10] Ang-Tiu CU, Meghrajani CF, Maano CC (2012). Pimecrolimus 1% cream for the treatment of seborrheic dermatitis: a systematic review of randomized controlled trials. *Expert Review of Clinical Pharmacology*.

[11] McCaughey C, Machan M, Bennett R, Zone JJ, Hull CM (2010). Pimecrolimus 1% cream for oral erosive lichen planus: a 6-week randomized, double-blind, vehicle-controlled study with a 6-week open-label extension to assess efficacy and safety. *Journal of the European Academy of Dermatology and Venereology*.

[12] Lebwohl M, Freeman AK, Chapman MS, Feldman SR, Hartle JE, Henning A (2004). Tacrolimus ointment is effective for facial and intertriginous psoriasis. *Journal of the American Academy of Dermatology*.

[13] Gorantla VS, Barker JH, Jones JW, Prabhune K, Maldonado C, Granger DK (2000). Immunosuppressive agents in transplantation: mechanisms of action and current anti-rejection strategies. *Microsurgery*.

[14] Truchuelo T, Díaz-Ley B, Ríos L, Alcántara J, Jaén P (2012). Facial angiofibromas treated with topical rapamycin: an excellent choice with fast response. *Dermatology Online Journal*.

[15] Ormerod AD, Shah SAA, Copeland P, Omar G, Winfield A (2005). Treatment of psoriasis with topical sirolimus: preclinical development and a randomized, double-blind trial. *British Journal of Dermatology*.

[16] Soria A, Agbo-Godeau S, Taïeb A, Francès C (2008). Treatment of refractory oral erosive lichen planus with topical rapamycin: 7 Cases. *Dermatology*.

[17] Allison AC, Eugui EM (1993). Immunosuppressive and other effects of mycophenolic acid and an ester prodrug, mycophenolate mofetil. *Immunological Reviews*.

[18] Bertelmann E, De Ruijter M, Gong N, Knapp S, Pleyer U (2009). Survival of corneal allografts following topical treatment with the immunomodulator mycophenolate mofetil. *Ophthalmologica*.

[19] Shoji Y, Fukumura T, Kudo M, Yanagawa A, Shimada J, Mizushima Y (1994). Effect of topical preparation of mycophenolic acid on experimental allergic contact dermatitis of guinea-pigs induced by dinitrofluorobenzene. *Journal of Pharmacy and Pharmacology*.

[20] Prausnitz MR, Langer R (2008). Transdermal drug delivery. *Nature Biotechnology*.

[21] Williams AC, Barry BW (2004). Penetration enhancers. *Advanced Drug Delivery Reviews*.

[22] Kalia YN, Naik A, Garrison J, Guy RH (2004). Iontophoretic drug delivery. *Advanced Drug Delivery Reviews*.

[23] Prausnitz MR (2004). Microneedles for transdermal drug delivery. *Advanced Drug Delivery Reviews*.

[24] Hautz T, Zelger B, Grahammer J (2010). Molecular markers and targeted therapy of skin rejection in composite tissue allotransplantation. *American Journal of Transplantation*.

[25] Schön MP, Krahn T, Schön M (2002). Efomycine M, a new specific inhibitor of selectin, impairs leukocyte adhesion and alleviates cutaneous inflammation. *Nature Medicine*.

[26] Gruber SA (1992). The case for local immunosuppression. *Transplantation*.

[27] Gruber SA, Hrushesky WJM, Cipolle RJ (1989). Local immunosuppression with reduced systemic toxicity in a canine renal allograft model. *Transplantation*.

[28] Gruber SA, Cipolle RJ, Canafax DM (1988). An implantable pump for intrarenal infusion of immunosuppressants in a canine autotransplant model. *Pharmaceutical Research*.

[29] Ruers TJM, Buurman WA, Smits JFM (1986). Local treatment of renal allografts, a promising way to reduce the dosage of immunosuppressive drugs. Comparison of various ways of administering prednisolone. *Transplantation*.

[30] Yano K, Fukuda Y, Sumimoto R, Sumimoto K, Ito H, Dohi K (1994). Suppression of liver allograft rejection by administration of 15-deoxyspergualin. Comparison of administration via the hepatic artery, portal vein, or systemic circulation. *Transplant International*.

[31] Stepkowski SM, Goto S, Ito T (1989). Prolongation of heterotopic heart allograft survival by local delivery of continuous low-dose cyclosporine therapy. *Transplantation*.

[32] Wang X, Alfrey EJ, Posselt A, Tafra L, Alak AM, Dafoe DC (1995). Intraportal delivery of immunosuppression to intrahepatic islet allograft recipients. *Transplant International*.

[33] Iacono AT, Keenan RJ, Duncan SR (1996). Aerosolized cyclosporine in lung recipients with refractory chronic rejection. *American Journal of Respiratory and Critical Care Medicine*.

[34] Iacono AT, Smaldone GC, Keenan RJ (1997). Dose-related reversal of acute lung rejection by aerosolized cyclosporine. *American Journal of Respiratory and Critical Care Medicine*.

[35] Iacono AT, Johnson BA, Grgurich WF (2006). A randomized trial of inhaled cyclosporine in lung-transplant recipients. *The New England Journal of Medicine*.

[36] Deuse T, Blankenberg F, Haddad M (2010). Mechanisms behind local immunosuppression using inhaled tacrolimus in preclinical models of lung transplantation. *American Journal of Respiratory Cell and Molecular Biology*.

[37] Furtado A, Perdigoto R, Oliveira F (2000). Local immunosuppression in clinical small bowel transplantation (report of two cases). *TPS*.

[38] Swann G (2010). The skin is the body's largest organ. *Journal of Visual Communication in Medicine*.

[39] Jensen JM, Proksch E (2009). The skin's barrier. *Giornale Italiana di Dermatologia. Minerva Dermatologica*.

[40] Bos JD (1997). The skin as an organ of immunity. *Clinical & Experimental Immunology*.

[41] Cendales LC, Kirk AD, Moresi JM, Ruiz P, Kleiner DE (2006). Composite tissue allotransplantation: classification of clinical acute skin rejection. *Transplantation*.

[42] Kanitakis J, Jullien D, Petruzzo P (2003). Clinicopathologic features of graft rejection of the first human hand allograft. *Transplantation*.

[43] Mathes DW, Randolph MA, Solari MG (2003). Split tolerance to a composite tissue allograft in a swine model. *Transplantation*.

[44] Schneeberger S, Gorantla VS, Hautz T, Pulikkottil B, Margreiter R, Lee WPA (2009). Immunosuppression and rejection in human hand transplantation. *Transplantation Proceedings*.

[45] Siemionow M, Ozturk C (2012). Face transplantation. *Journal of Craniofacial Surgery*.

[46] Ravindra KV, Buell JF, Kaufman CL (2008). Hand transplantation in the United States: experience with 3 patients. *Surgery*.

[47] Morelon E, Kanitakis J, Petruzzo P (2012). Immunological issues in clinical composite tissue allo. *Transplantation, Transplantation*.

[48] Siemionow M, Ozturk C (2011). An update on facial transplantation cases performed between 2005 and 2010. *Plastic and Reconstructive Surgery*.

[49] Reitamo S, Rissanen J, Remitz A (1998). Tacrolimus ointment does not affect collagen synthesis: results of a single-center randomized trial. *Journal of Investigative Dermatology*.

[50] Banchereau J, Steinman RM (1998). Dendritic cells and the control of immunity. *Nature*.

[51] Wollenberg A, Sharma S, Von Bubnoff D, Geiger E, Haberstok J, Bieber T (2001). Topical tacrolimus (FK506) leads to profound phenotypic and functional alterations of epidermal antigen-presenting dendritic cells in atopic dermatitis. *Journal of Allergy and Clinical Immunology*.

[52] Panhans-Groß A, Novak N, Kraft S, Bieber T (2001). Human epidermal Langerhans' cells are targets for the immunosuppressive macrolide tacrolimus (FK506). *Journal of Allergy and Clinical Immunology*.

[53] Hoetzenecker W, Ecker R, Kopp T, Stuetz A, Stingl G, Elbe-Bürger A (2005). Pimecrolimus leads to an apoptosis-induced depletion of T cells but not Langerhans cells in patients with atopic dermatitis. *Journal of Allergy and Clinical Immunology*.

[54] Caproni M, Torchia D, Schincaglia E (2006). Expression of cytokines and chemokine receptors in the cutaneous lesions of erythema multiforme and Stevens-Johnson syndrome/toxic epidermal necrolysis. *British Journal of Dermatology*.

[55] Shirbacheh MV, Ren X, Jones JW (1999). Pharmacokinetic advantage of intra-arterial cyclosporin A delivery to vascularly isolated rabbit forelimb. I. Model development. *Journal of Pharmacology and Experimental Therapeutics*.

[56] Shirbacheh MV, Harralson TA, Jones JW (1999). Pharmacokinetic advantage of intra-arterial cyclosporin A delivery to vascularly isolated rabbit forelimb. II. Dose dependence. *Journal of Pharmacology and Experimental Therapeutics*.

[57] Shirbacheh MV, Jones JW, Harralson TA (1999). Pharmacokinetics of intra-arterial delivery of tacrolimus to vascularly isolated rabbit forelimb. *Journal of Pharmacology and Experimental Therapeutics*.

[58] Shirbacheh MV, Jones JW, Breidenbach WC, McCabe S, Barker JH, Gruber SA (1998). The case for local immunosuppression in composite tissue allotransplantation. *Transplantation Proceedings*.

